# Acceptability of minitablets in soft food. A randomised cross-over study in children 

**DOI:** 10.3389/fphar.2025.1702183

**Published:** 2026-01-05

**Authors:** Jennifer C. Duncan, Rebecca Page, Janet Clark, Gabrielle Seddon, Joy Slater, Andrea Gill, Silothabo Dliso, Udeme Ohia, Nikolaos Skoutelis, Leonie Wagner-Hattler, Adrian Baumgartner, Angela Sprunk, Peter Kühl, Louise Bracken

**Affiliations:** 1 Paediatric Medicines Research Unit (PMRU), Alder Hey Children’s NHS Foundation Trust, Liverpool, United Kingdom; 2 NIHR Alder Hey Clinical Research Facility, Alder Hey Children’s NHS Foundation Trust, Liverpool, United Kingdom; 3 Department of Paediatric Nephrology, Alder Hey Children’s NHS Foundation Trust, Liverpool, United Kingdom; 4 Pharmaceutical R&D, F. Hoffmann-La Roche Ltd., Basel, Switzerland

**Keywords:** acceptability, swallowability, palatability, composite endpoint on acceptability, paediatric formulations, minitablets

## Abstract

**Objective:**

To evaluate the acceptability of placebo minitablets mixed with different volumes of soft food in children aged 6 months to 7 years.

**Methods:**

In this UK hospital-based, randomised cross-over study, children received placebo minitablets in yoghurt or apple sauce. Those under 1 year received one sample; older children received two. All participants were video recorded taking the samples. Minitablet counts (50–135), and soft food volumes (7.5–30 mL) increased with age. Children aged 1 year + were randomised to “high” or “low” soft food volumes for the first sample; parents chose the soft-food amount for younger children. Swallowability was rated (1–5) by researchers, and palatability by independent reviewers (‘pleasant’, ‘neutral’, or ‘unpleasant’). Palatability and swallowability scores were then combined to assess overall acceptability using the composite endpoint tool. Children aged 4–7 years completed a participant questionnaire.

**Results:**

100 children were grouped by age: <2 years (G1, n = 16), 2–4 years (G2, n = 37), and ≥5 years (G3, n = 47). Mean age was 4.2 years; 56% were male; 84% were tablet naïve. Youngest was 9 months old. Yoghurt was preferred by 84%. Swallowability was 77% overall, increasing with age (G1: 69%, G2: 73%, G3: 83%). G3 participants consumed more per sample than G1 (at least 80% of minitablets consumed for Samples 1/2, respectively: G3-89%/91% vs. G1-69%/62%). ‘Pleasant’ was the most common palatability rating (48% Sample 1, 52% Sample 2). Some older children reported finding the number of minitablets excessive. Acceptability was 46% (G1), 58% (G2), and 53% (G3), with overall acceptability rated as “high/good” for 54% of participants.

**Conclusion:**

Minitablets in soft food were generally acceptable for children aged 9 months to 7 years, especially those aged 2–4 years. Swallowability and palatability were good across all age groups. Larger soft-food volumes were often preferred, but both volumes were well tolerated.

## 1 Introduction

Medicines should be easy to use and acceptable to the intended users (European Medicines Agency, 2013). In the case of children, evaluating acceptability is a key part of developing effective paediatric medicines. Involving the target age group in the design process helps ensure the final product is appropriate for a broad range of users (European Medicines Agency, 2013; Lajoinie et al., 2017). Many active pharmaceutical ingredients (APIs) have a bitter taste, and children are naturally more sensitive to bitterness and texture differences (such as grittiness) than adults. This heightened sensitivity can negatively affect a child’s willingness to take the medicine, ultimately impacting treatment adherence (Meruva et al., 2024; Lura et al., 2025).

Minitablets (MTs) are small tablets, generally 4 mm or less in diameter, designed for single or multiple-unit dosing and are often suitable for sprinkling onto soft foods (Meruva et al., 2024; Lura et al., 2025). According to U.S. Food and Drug Administration (FDA) guideline, the recommended bead size for sprinkle formulations is up to 2.5 mm, with an upper limit of 2.8 mm (Food and Drug Administration FDA, 2012).

MTs are increasingly being recognised as a potential alternative to oral liquid medicines, especially for paediatric use (Aleksovski et al., 2014). While oral liquids offer the advantages of easy swallowing and flexible dosing, they are often associated with several limitations, including unpleasant taste and texture, less desirable excipient profiles, and the requirement for dose measurement. This last factor can lead to administration errors, particularly when different concentrations of the same API are available (Conn et al., 2019).

MTs combine the benefits of traditional solid oral dosage forms (SODFs) - including stability, ease of storage and transport, precision dosing, cost efficiency, and effective taste masking - with the added advantage of being small enough to swallow easily (Litalien et al., 2022). They can also be mixed with soft foods to facilitate administration. However, their small size limits the amount of active ingredient they can contain, often requiring multiple tablets per dose (Lopez et al., 2015). As a result, a wide range of strengths may be necessary to accommodate the dose load and varying needs of paediatric patients.

Several studies have demonstrated that the administration of single MTs is well tolerated by young children, including neonates (Thomson et al., 2009; Van de Vijver et al., 2011; Spomer et al., 2012; Klingmann et al., 2013; Klingmann et al., 2015; Van Riet-Nales et al., 2013). Additional paediatric studies have explored the acceptability of administering multiple MTs, with doses ranging from 4 to 400 MTs being assessed (Kluk et al., 2015; Klingmann et al., 2018; Mitsui et al., 2022; Münch et al., 2023; Klingmann et al., 2023). A 2018 study by Klingmann et al. found that administering either 25 or 100 MTs (in soft food or with a drink) was better accepted and easier to swallow, compared to an equivalent dose of oral syrup (5–10 mL) among 372 children aged 6 months to 5 years. However, when 400 MTs were administered to children aged 2 years and older, the formulation was deemed unacceptable (Klingmann et al., 2018). The ideal number of MTs that can be safely and comfortably taken daily by children on long-term treatment remains unknown.

Currently, there is no standardised methodology for assessing the acceptability of paediatric formulations, and the European Medicines Agency (EMA) has not endorsed any specific assessment tool. The Composite Endpoint (CEP) tool (Wargenau et al., 2022) was developed as a structured and standardised approach to evaluate the acceptability of oral paediatric formulations by combining measures of swallowability and palatability. Although the EMA issued a Letter of Support (European Medicines Agency, 2023), a formal Qualification Opinion has not been granted, indicating that further development and validation are required before regulatory endorsement can be achieved.

The CEP tool may be applied in two proposed key contexts: (i) “At an early stage, by testing placebo formulations to identify the most suitable formulation principles for children in the respective age ranges,” and (ii) “As a secondary endpoint to confirm the acceptability of the active formulation, e.g., within a safety and efficacy trial for a new medicine in development agreed upon within a paediatric investigation plan” (European Medicines Agency, 2023). While internal validation has been conducted using an adapted version of the CEP tool (Münch et al., 2024), external validation in different settings is required.

This study, CAMEO (Children’s Acceptability of Minitablets mixEd with fOod) used the CEP tool to evaluate the acceptability of placebo MTs in children. Specifically, it assessed how easily the tablets could be swallowed (swallowability) and how they were perceived in terms of taste and mouthfeel (palatability) when mixed into a preferred soft food. It also aimed to establish the minimum amount of soft food that could be combined with a fixed number of MT placebos, while remaining acceptable to children as a single dose.

## 2 Materials and methods

### 2.1 Study design

**FIGURE 1:**
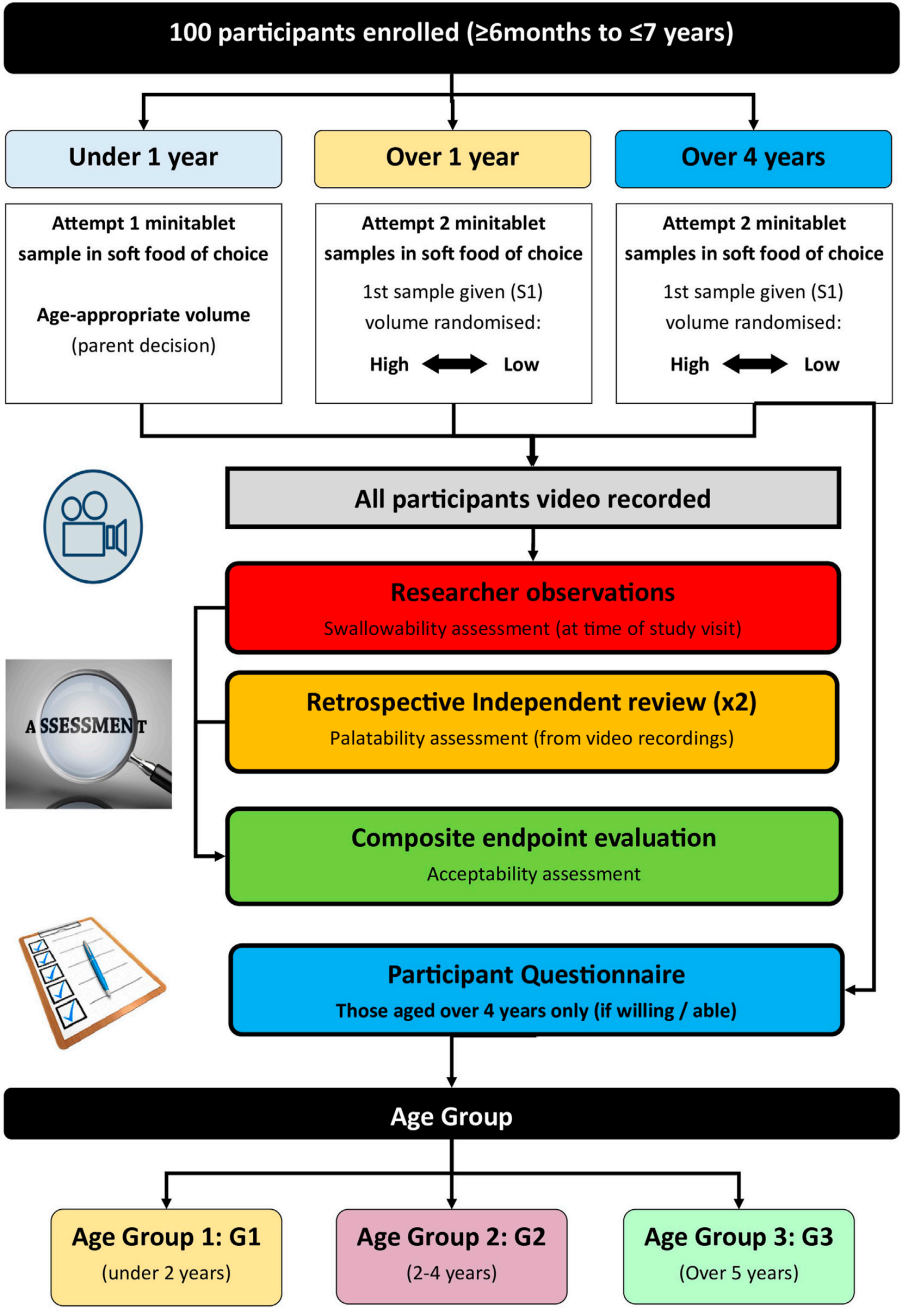
CAMEO study design.

A randomised cross-over study to investigate the acceptability of placebo MTs when incorporated into different volumes of soft food for children aged 6 months to 7 years old. The CEP for ‘acceptability’ combines swallowability and palatability outcomes for each MT sample attempted. The study design is shown in Figure 1.

#### 2.1.1 Setting

The study took place on National Institute for Health Research (NIHR) clinical research facility (CRF) ward at Alder Hey Children’s NHS Foundation Trust (AH).

#### 2.1.2 Patient and public involvement

Children and young people from the Liverpool Young Persons’ Advisory Group (YPAG) contributed feedback on the design of previous similar acceptability studies conducted by the Paediatric Medicines Research Unit (PMRU) team. Additionally, a parent from the Liverpool YPAG reviewed and approved the study information leaflets for children aged 4–5 and 6–7 years and supported the proposed method of MT administration used in this study.

#### 2.1.3 Participant identification and consent

Children aged 6 months to 7 years were recruited between September 2023 and March 2024 via the hospital, either as NHS patients (inpatient/outpatient) or visitors (e.g., staff children). All participants were considered ‘healthy volunteers’. Recruitment was opportunistic and supported through hospital adverts, emails, the intranet, posters in outpatient areas, and promotion by Parent Champions in local community health centres. A target of at least 10–20 participants was set for each age group (G1, G2, and G3).

An age-appropriate participant information sheet was provided to each child and their parent/legal guardian, who were given time to review the materials and ask questions. Informed consent was obtained from the parent/guardian, and assent (verbal or written) was sought from children aged 6 and above when possible. Participants agreed to be video recorded while attempting to swallow each sample; recordings were deleted at study closure.

Eligibility was confirmed by the research team, including risk assessment and checks for allergies or swallowing concerns. Participants were required to have been weaned onto a variety of soft foods for at least 4 weeks before enrolment (Table 1). Each participant received a £25 voucher as a goodwill gesture to acknowledge their time, and any costs incurred (e.g., parking).

**TABLE 1:** Eligibility criteria.

Inclusion criteria	Exclusion criteria
Children aged ≥6 months to ≤7 years	Children aged <6 months or ≥8 years
No restriction on nationality, ethnicity, or socio-economic status	Parent/legal guardian/child unable to understand study information in English and declines language line support
Minitablets suitable for vegetarians/vegans	Known allergy/intolerance to minitablet ingredients or soft foods
Dairy-intolerant/vegan participants may provide a suitable yoghurt alternative (if requested)	Child unwilling to take soft food samples
Verbal assent from 6–7-year-olds. Written assent also requested (if able/willing)	Child aged ≥6 years does not provide verbal assent
Written informed consent from parent/legal guardian	Parent/carer unable or unwilling to provide written consent
Ability to swallow: No clinical/parental concerns about swallowing	Impairment of swallowing or conditions affecting safe swallowing
Willingness for video recording by child/parent/legal guardian	Current illness affecting taste or smell perception
Parent confirms eligibility	Pronounced family distress or child protection concerns
No prior experience of taking tablet(s) needed	Child not weaned onto a range of soft foods for at least 4 weeks prior to enrolment

#### 2.1.4 Intervention - minitablet formulation

**FIGURE 2:**
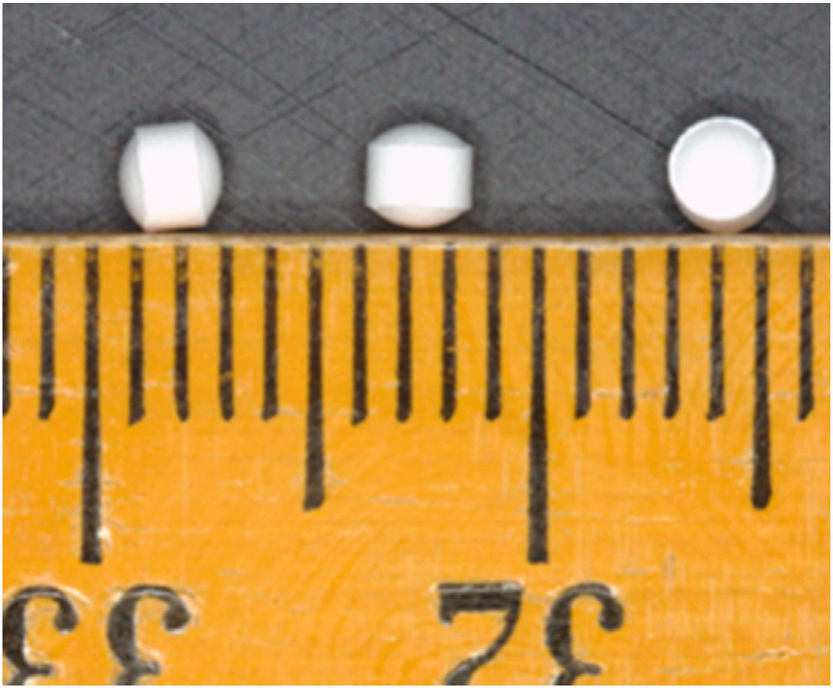
The CAMEO study minitablets, measuring 2.3 mm in diameter.

Placebo, white, round biconvex coated (OPADRY® II White), flavourless MTs, measuring 2.3 mm in diameter (average weight 10.5 mg) were used in this study (Figure 2). The MT formulation consisted of isomalt, microcrystalline cellulose, croscarmellose sodium and sodium stearyl fumarate. The MTs were manufactured to good manufacturing practice standards by F. Hoffmann-La Roche Ltd.

Participants were required to attempt one or two MT samples, depending on their age. The number of placebo MTs contained within each sample was fixed, ranging from 50 to 135 MTs dependent on the age of the participant (Table 2), reflecting the real-life dosing range of an active drug for this paediatric age group. MT samples were pre-dispensed by Researcher 2 (R2) into standard amber PET tablet bottles fitted with child-resistant click-lock caps, using a minitablet counting device capable of dispensing up to 24 MTs at a time. All bottles were appropriately labelled. The first sample (S1) was randomised to either the ‘high’ or ‘low’ soft food volume using a pre-generated randomisation list (www.sealedenvelope.com/simple-randomiser/v1/lists). The MTs were then incorporated into an ‘age-appropriate’ amount of soft food of choice for children under 1 year, or into either a ‘high’ or ‘low’ volume for children aged over 1 year (Table 2). The second sample (S2) represented the alternate soft food volume, as specified by the same randomisation list.

**TABLE 2:** Number of coated minitablets given per sample based on CAMEO participant’s age.

Age group	Age range	Number of minitablets (fixed)	Minitablet volume (cm^3^)	Soft food amount -single sample only		Total volume of soft food (1 sample)	Soft food of choice (SFOC)
Under 1 year	6 to <12 months	50	0.38	Given in an age-appropriate volume (no set minimum or maximum amount)		As per parental choice	Apple sauce or smooth flavoured yoghurt

Age group	Age range	Number of minitablets (fixed)	Minitablet volume (cm^3^)	Soft food amount - 1st sample randomised		Total volume of soft food (2 samples - approx)	Soft food of choice (SFOC)^a^
				Low volume (approx)	High volume (approx)		
Over 1 year	1 to <2 years	70	0.54	7.5 mL	15 mL	22.5 mL	Apple sauce or smooth flavoured yoghurt^b^
	2 to <4 years	95	0.72	10 mL	20 mL	30 mL	
	4 to ≤5 years	115	0.88	12.5 mL	25 mL	37.5 mL	
	6 to ≤7 years	135	1.04	15 mL	30 mL	45 mL	

^a^ SFOC, selected must be the same for both samples attempted.

^b^ For dairy intolerant/vegan participants only: A dairy-free (DF), smooth yoghurt could be supplied by the parent if the child did not wish to take the minitablet sample(s) in apple sauce. DF brand/flavour used during the study was documented.

Study visits were conducted by two researchers: Researcher 1 (R1) obtained consent and led the visit, while the second researcher prepared MT samples and recorded observations.

MTs were gently stirred into the soft food of choice (strawberry yoghurt or apple sauce). Each sample was freshly prepared, and immediately offered to the child to try, using a spoon. The researcher did not instruct the participant ‘not to chew’ the MT sample(s) unless they specifically asked this question prior to, or during the administration period. The participant’s mouth was visually inspected after each sample attempt, using a pen torch, if the child allowed. Both participants and R1 were blinded as to which soft food volume (high or low) was provided first. A 5-minute break was offered between samples, if needed. Participation was voluntary, and families could withdraw at any time without explanation.

#### 2.1.5 Participant questionnaires

**FIGURE 3:**
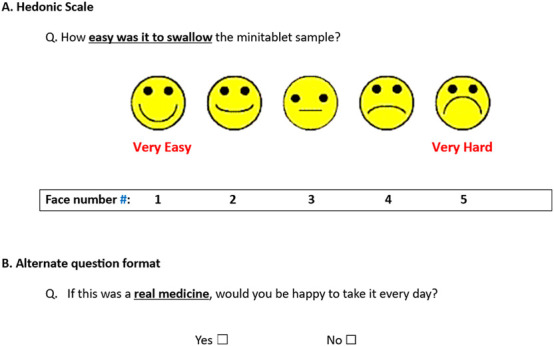
Sample question(s) from the CAMEO participant questionnaire - **(A)** Hedonic Scale and **(B)** Alternate question format # Face numbers were not displayed on the questionnaire completed by participants.

All participants were video recorded whilst attempting to swallow the MT sample(s), solely for assessment purposes. After each attempt, participants aged 4 years and older were asked to complete a short paper-based questionnaire. These were filled out by the children themselves, either independently or with assistance from a parent/carer, or researcher R1. The MT samples were rated using a 5-point facial hedonic scale, as illustrated in Figure 3A with anchors labelled as ‘very easy’ and ‘very hard’ in this example. Responses were considered positive if participants selected faces 1 to 3, indicating a positive to neutral experience. Additional closed response questions (i.e., yes/no) were also included, with an example shown in Figure 3B. When two samples were attempted, participants were also asked to indicate which sample they preferred based on how easy it was to swallow. Participants were not obligated to answer all questions if they preferred not to respond.

#### 2.1.6 Researcher observations

Researcher observations were carried out for all participants, with assessments of both swallowability and palatability conducted for each sample attempted. General background information was also recorded by R1, including current medical history, frequency of prescribed medication use (e.g., daily or only when unwell), types of previously used medications (e.g., oral liquids, inhalers), any prior experience with taking tablets (regardless of size or shape) and the type of soft food chosen by the parent, child, or both.

Swallowability was assessed on the day of the study visit by R2 using a 5-point scale, where a score of 1 indicated the sample was ‘completely swallowed’ and a score of 5 indicated ‘refusal or ingestion of less than 80% of the total number of MTs offered’.

Prior to conducting the palatability assessments, the independent reviewers (UO and NS) participated in a 1-hour training session delivered by LB and AG. The session included the scoring of example videos demonstrating a range of palatability outcomes and allowed reviewers to clarify any aspects of the assessment procedure. Palatability was subsequently evaluated independently by the two reviewers, who retrospectively examined the study video recordings and rated participant responses using a 3-point scale (1 = pleasant, 2 = neutral, 3 = unpleasant). In cases of extreme disagreement - defined as a difference of more than one scale point (e.g., pleasant vs. unpleasant) - the final rating was determined by the Chief Investigator (CI, LB), who was not involved in data collection.

Acceptability was then determined using the CEP tool (Supplementary Tables S1–4, adapted/reproduced from (Wargenau et al., 2022)), which combines researcher-rated swallowability and palatability scores to generate an overall rating ranging from “highly acceptable” to “not acceptable”. If a video recording was unavailable, palatability (and therefore CEP scores) could not be calculated.

### 2.2 Data analysis

Data were grouped into three age cohorts (G1, G2 and G3) and summarised using descriptive statistics, including mean, median, range, counts, and percentage. The study was exploratory and not powered for statistical comparisons, so no formal hypothesis testing was performed. Reasons for refusal and any additional participant feedback were documented.

## 3 Results

### 3.1 Demographics

A total of 100 children were enrolled in the study (98 visitors and 2 NHS outpatients), ranging in age from 9 months to 7 years, with a median age of 4 years and a mean age of 4.2 years. Children aged 7 years represented the largest proportion of participants (18%, Supplementary Figure S5). For analysis, participants were categorised into three broader age groups, resulting in uneven group sizes (Table 3).

**TABLE 3:** Participant demographics.

Outcome		Age group		
		G1^a^(<2** **years old)	G2 (2 to ≤4** **years old)	G3 (≥5** **years old and over)
Number of participants (n =)		16	37	47
Sub-age Group (n =)	Under 1 year (asked to try one sample)	3		
	Over 1 year (asked to try two samples)	97		
Breakdown by age (in years)	Median	1	3	6
	Mean	0.96	3.08	6.06
	Mode	1	3	7
Gender	Male	5	20	30
	Female	11	17	16
	Unknown	0	0	1
Tablet naivety (n =)		100% (16)	97% (36)	68% (32)
How often do you use/take medicines?	Everyday	9		
	When poorly	91		
Currently taking any prescribed medicines?	Yes	15		
	No	85		
Researcher observations completed		100		
Visual inspections conducted (S1 v S2)		67% / 78%	89% / 100%	100% / 100%
Refusals (S1 v S2, 12 in total - S1 = 1, S2 = 11)		6% / 25%	0% / 14%	0% / 7%
Participant questionnaires (over 4’s only)		N/A	60	

^a^ Includes 3 participants who only attempted one sample.

S1 = Sample 1 (1st sample given), S2 = Sample 2 (2nd sample given).

### 3.2 Soft food choice, sample administration and administration time

Yoghurt was the preferred soft food choice for most participants (84%). For children under 1 year, parents administered the MTs, while 58% of children over 1 year self-administered. The average administration time was 3:49 minutes for those under 1, compared to 2:12 minutes (Sample 1) and 1:44 minutes (Sample 2) for older children.

### 3.3 Swallowability

Table 4 shows that swallowability was high, with 77% of participants fully consuming all MT samples they attempted. Swallowability increased with age, ranging from 69% in G1 to 83% in G3.

**TABLE 4:** CAMEO study researcher observations by age group.

Outcome		Age group						Total
		G1^a^ (<2** **years old)		G2 (2 to ≤4** **years old)		G3 (≥5** **years old and over)		
		S1	S2	S1	S2	S1	S2	
Total number of participants		16		37		47		100
Participant swallowability (full intake of all minitablet samples offered*)		69% (11)		73% (27)		83% (39)		77% (77)
Total number of samples offered per group		29		74		94		197
Refusals (per sample)		0	4	1	5	0	2	12
≥80% of the minitablets consumed per sample (n =)		69% (11)	62% (8)	84% (31)	78% (29)	89% (42)	91% (43)	83% (164∼)
Swallowability scores (n =)	Completely swallowed (score 1)	6	4	9	9	11	16	55
	Partially swallowed (at least 80% of sample taken) (Score 2)	3	3	22	20	31	27	106
	Spat out (score 3)	0	0	1	1	1	0	3
	Swallowed the wrong way (score 4)	3∼	1∼	0	0	0	0	4∼
	Refused to take (or less than 80% of sample taken) (score 5)	4	5	5	7	4	4	29
Chewing (% of all samples) per age-group	Yes	21% (6)		53% (39)		50% (47)		47% (92)
	No	66% (19)		39% (29)		48% (45)		47% (93)
	N/A (refusal)	14% (4)		8% (6)		2% (2)		6% (12)

S1 = Sample 1 (1st sample given), S2 = Sample 2 (2nd sample given).

^a^ Participants in G1 under 1** **year old were given one sample only.

∼ Three G1 participants consumed over 80% of the sample(s) but received a score of 4 (cough).

Of all MT samples attempted, the majority (161 out of 197; 82%) received a swallowability score of 1 or 2, based on whether the sample was chewed and/or if any residues were observed in the mouth during visual inspection (Table 4).

### 3.4 Amount of minitablets consumed, chewing and refusals

Similar consumption rates (Swallowability score of 1 or 2 = ≥80% of the number of MTs provided were swallowed) were achieved across all age groups for both samples. Across all three age groups, 84% (84/100) participants managed to consume 80% or more of their first (or single) sample, whereas 82% (80/97) participants successfully consumed this amount (or more) of their second sample. However, 11 participants immediately refused (did not attempt) their second sample. One participant refused to take both samples provided (Table 4).

Chewing was observed across all age groups and seen in 47% (92/197) of sample administrations (Table 4).

### 3.5 Palatability

Eleven palatability scores could not be calculated due to the following reasons: no video - refused to attempt second sample/activity stopped (n = 4), video available - sample offered and immediately refused (n = 4) and single sample (n = 3). Percentage exact agreement (%EA) between the two independent reviewers was initially found to be 53% (105/200, S1 = 60/100 and S2 = 45/100). Due to extreme disagreement, 23 study videos (9 for S1 cases and 14 for S2 cases) were reassessed by the study CI. Table 5 shows the most common outcome for palatability across all age groups was ‘pleasant’ for both samples at 51% (100/197).

**TABLE 5:** Composite endpoint results for overall acceptability per age group.

Outcome		Age group			Total
		G1^a^ (<2** **years old)	G2 (2 to ≤4** **years old)	G3 (≥5** **years old and over)	
Swallowability scores (n = 197)	Completely swallowed (score 1)	10	18	27	55
	Partially swallowed (at least 80% of sample taken) (Score 2)	6	42	58	106
	Spat out (score 3)	0	2	1	3
	Swallowed the wrong way (score 4)	4∼	0	0	4∼
	Refused to take (or less than 80% of sample taken) (score 5)	9	12	8	29
Combined rater palatability (#) (n = 197)	Pleasant (score 1)	15	38	47	100
	Neutral (score 2)	1	7	12	20
	Unpleasant (score 3)	10	26	33	69
	N/A (immediate refusal)	3	3	2	8
Acceptability via CEP assessment per age-group (#) (n = 197)	Highly acceptable (high)	9	11	18	38
	Good acceptability (good)	3	30	31	64
	Low acceptability (low)	2	8	16	26
	Not acceptable (no)	12	22	27	61
	N/A- unable to CEP score (immediate refusal)	3	3	2	8
Overall acceptability (n = 189) CEP = high/good, when all ‘unassessable samples’ removed		46% (12)	58% (41)	53% (49)	54% (102)

S1 = Sample 1 (1st sample given), S2 = Sample 2 (2nd sample given).

CEP, composite endpoint, (#) following CI review.

^a^ Participants in G1 under 1 year old were given one sample only.

∼ Three G1 participants consumed over 80% of the sample(s) but received a score of 4 (cough).

### 3.6 Composite endpoint assessment for acceptability

Out of 189 MT samples attempted and where videos were available for review purposes, 54% (102/189) of the MT sample administrations were determined to have “high” or “good” acceptability using the CEP tool therefore deemed ‘acceptable’ to the participants. Acceptability varied across the age groups from 46%, 58% and 53%, respectively, with G2 participants (2–4 years) demonstrating the highest acceptability (Table 5).

### 3.7 High v low volume of soft food

86 participants (86%) attempted both MT samples offered (Table 6). Each child had either the ‘high volume’ sample followed by ‘low volume’ sample of soft food, or vice versa, depending on the randomisation sequence assigned at time of recruitment.

**TABLE 6:** Acceptability by soft food volume.

Soft food sample sequence	High volume acceptable	Low volume acceptable	Both acceptable	Both not acceptable	Total
High then low volume	8	8	18	10	44
Low then high volume	7	5	18	12	42
Total	15	13	36	22	86

Participants were excluded from this data analysis as follows: Only offered one sample as under 1 years old (n = 3) and Child refused second sample (or both samples) (n = 11). Acceptable = ‘high’ or ‘good’ CEP outcome result.

### 3.8 Participant reported outcomes

A total of 60 participants aged 4 years and older (G2: n = 13; G3: n = 47) completed the participant questionnaire. Of these, the majority - 65% (39 out of 60) - were completed with assistance from the researcher, 16 were completed independently by the children, 3 were completed with assistance from a parent, and 2 were completed collaboratively by the child, parent, and researcher.

**FIGURE 4:**
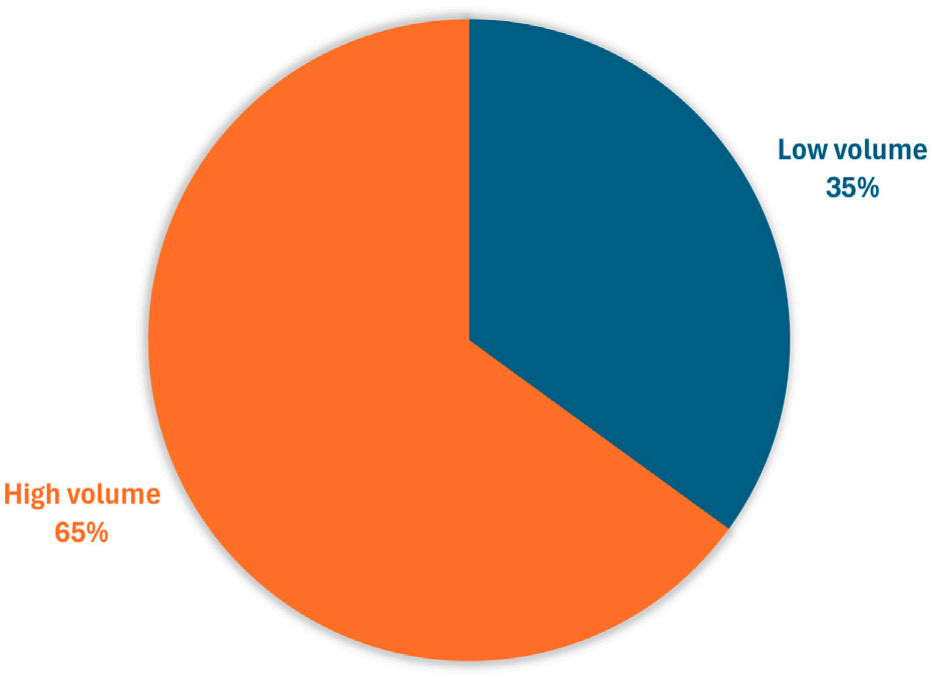
Participants’ preference for the easiest sample to swallow (n = 57*) *Three participants aged 4 years and over did not try both samples so were unable to rank.

Two participants did not answer some of the questions related to S1, leading to missing data (one related to a ‘high’ volume sample, and the other a ‘low’ volume sample). The high-volume sample was rated the easiest to swallow with 65% (37/57) participants ranking it as their preferred choice (Figure 4).

**FIGURE 5:**
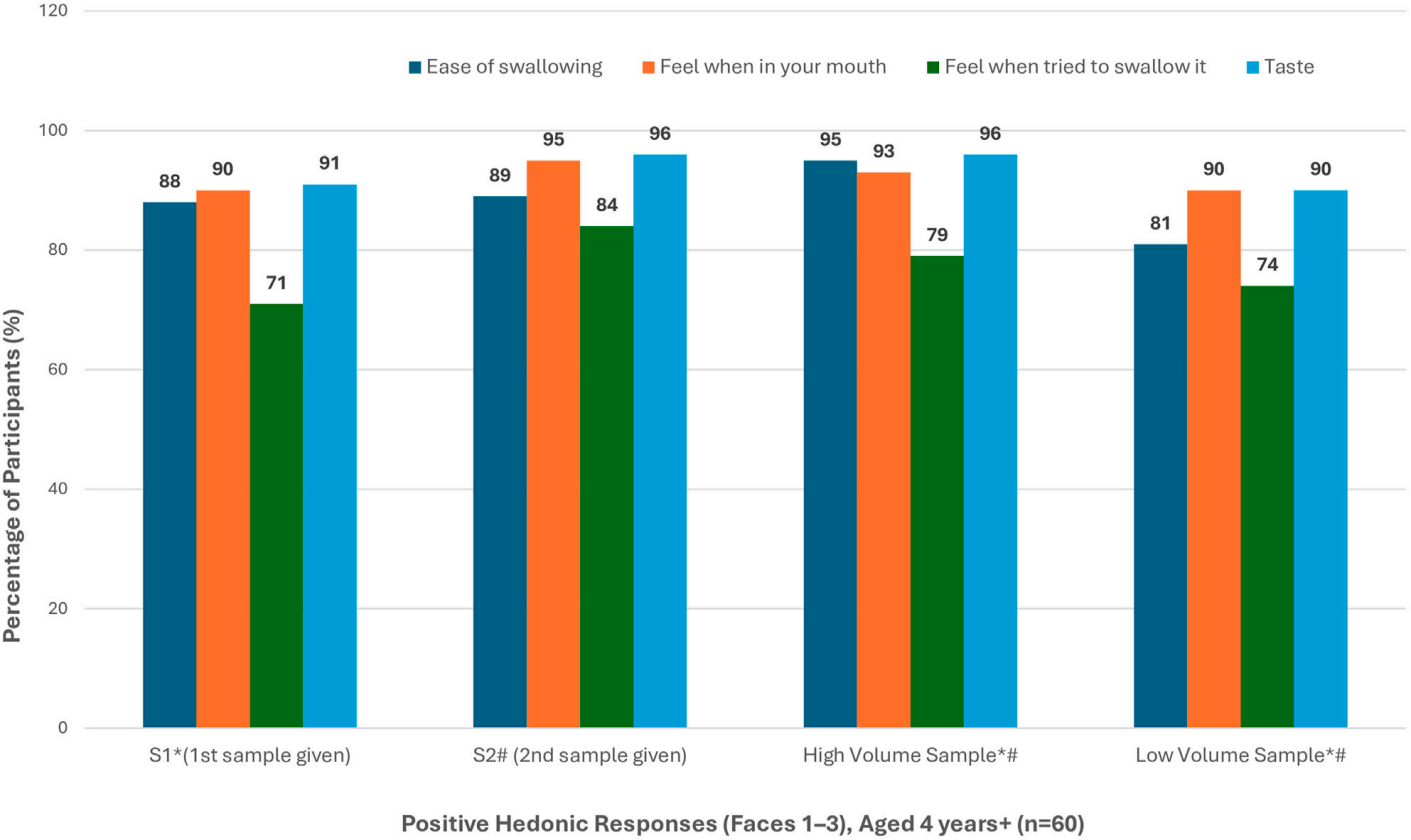
Positive hedonic response rates in participants aged 4 years and above (n = 60) * Missing data - Participant did not answer question (n = 2, 1 = “High” and 1 = “Low” - all first sample) # Study activity stopped - sample refused (n = 4, n = 2 refused “High” and n = 1 refused “Low” - all second sample).

Based on the total number of positive responses received on the 5-point facial hedonic scale (Faces 1–3), the ‘second’ and ‘high volume’ samples achieved the highest rating in terms of ease of swallowing, texture, and taste (Figure 5).

For S1, 69% (9/13) G2 participants reported that they would be “willing to take it every day”. This reduced to 46% (6/13) for their S2 administration. For G3 participants, 79% (37/47) reported that they would be “willing to take it every day” for S1. This reduced slightly to 74% (35/47) for their S2 administration.

### 3.9 Participant and parent feedback

Some participants and parents provided feedback on the acceptability of the MTs, with a selection of positive and negative quotes presented in Table 7.

**TABLE 7:** Participant and parent feedback on the placebo minitablet formulation.

Theme	Positive feedback	Negative feedback
Participant		
Taste	“Tastes nice.” CAM-G3-58, 5-year-old girl	“Tastes weird.” CAM-G3-87, 7-year-old girl
	“I loved the yoghurt.” CAM-G2-73, 4-year-old girl	“I bit one in half, and it didn't taste good. I Chewed some but I liked the apple sauce.” CAM-G3-92, 6-year-old girl
	“It’s tasty, good.” CAM-G3-42, 5-year-old boy	“Would be better if it was chocolate.” CAM-G3-21, 5-year-old boy
	“I’ve ate all the tablets. It was good. I Loved it. I Could eat a whole bowl of this if I wanted.” CAM-G3-42, 5-year-old boy	“The minitablets tasted like mints”. CAM-G3-68, 7-year-old boy
Mouthfeel	“Felt crunchy.” CAM-G3-81, 7-year-old boy	“Sample was same as first … bitty.” CAM-G3-02, 6-year-old girl
	“A bigger thumbs up - it is better. It is crunchy.” CAM-G2-59, 4-year-old boy	“Thumbs down. It felt lumpy.” CAM-G2-86, 4-year-old boy
Acceptability	“It was a good way to swallow tablets.” CAM-G3-15, 7-year-old girl	“I don't like the balls.” CAM-G2-89, 3-year-old girl
	“It will be a nice way to take medicine in the future.” CAM-G3-22, 6-year-old boy	“I didn't like it.” CAM-G3-100, 5-year-old girl
	“No preference, I liked both.” CAM-G2-95, 3-year-old girl	“I don't want anymore.” CAM-G2-94, 3-year-old girl
	“Thumbs up.” CAM-G2-59, 4-year-old boy	“Double thumbs down.” CAM-G2-60, 3-year-old boy
Parent		
Acceptability	“Easy to scoop.” Parent of CAM-G1-19, 14-month-old girl	“If the minitablets had flavoured the yoghurt, then they may not have completed the samples.” Parent of CAM-G2-78, 4-year-old girl
	“It’s a good way to take medicine although I was not sure if he would take it at first with it having bits in.” Parent of CAM-G2-55, 3-year-old boy	“Would have taken it if it wasn’t a smooth yoghurt.” Parent of CAM-G2-23, 2-year-old boy
	“I thought formulation was good/acceptable.” Parent of CAM-G1-70, 16-month-old girl	“He would usually eat smooth yoghurt, so I think it’s the bits that put him off eating it.” Parent of CAM-G2-66, 2-year-old boy
	“I thought it was fine.” Parent of CAM-G1-57, 13-month-old boy	“I don’t think she liked the apple sauce. I tried the apple sauce on its own, and I like it!” Parent of CAM-G1-41, 21-month-old girl
	“I think she could taste something else other than yoghurt, but it didn't bother her, she still ate it.” Parent of CAM-G1-61, 13-month-old girl	“I think it is because she can see the tablets that she doesn’t like it.” Parent of CAM-G3-100, 5-year-old girl
		“Dairy-free yogurt would be better.” Parent of CAM-G2-20, 2-year-old girl

## 4 Discussion

This single-centre, randomised cross-over study has demonstrated that placebo coated MTs, when co-administered with soft food, are an acceptable dosage form for healthy children. The study offers valuable insights and guidance for caregivers, healthcare providers and the pharmaceutical industry regarding age-appropriate administration of multiple coated MTs, in quantities ranging from 50 to 135 MTs per dose. Furthermore, it establishes a soft food volume range of 7.5 mL–30 mL that children aged 9 months to 7 years find acceptable. Incorporating such guidance into the Summary of Product Characteristics (SmPC) for relevant APIs in future could enhance acceptability and support more effective paediatric drug administration in real-life settings.

77% of participants fully consumed all MT samples they attempted; swallowability increased with age. Palatability was generally positive. Of the 197 cases evaluated from video recordings, more than half (51%) of all MT administrations were rated as ‘pleasant’ in terms of palatability, while 10% were deemed ‘neutral’. 35% were classified as ‘unpleasant’. 4% were unassessable.

Overall acceptability in our study was moderate, averaging 54% across age groups (46%–58%). In comparison, Münch et al. (2024) reported markedly higher CEP ratings for MTs, with acceptability of 93.8% in infants aged 1-<6 months, 88.9% in children aged 6-<12 years and 76.7% for 12-<18 years, likely reflecting differences in study populations. As the CEP tool does not define fixed thresholds for ‘success’ in overall acceptability or its components (e.g., swallowability and palatability), higher proportions of ‘highly acceptable’ or ‘good acceptability’ ratings generally indicate greater suitability of the formulation for children. In our study, the proportions of these positive ratings were 46%, 58%, and 53% across the three age groups (G1-G3), respectively. This level of acceptability was also lower than the 82.2% reported by Wargenau et al. (2022) for MTs. Notably, participants in G2 (2–4 years) demonstrated the highest acceptability in the CAMEO study. These findings underscore the importance of patient preference and dosage form flexibility.

As expected, all G1 participants had no prior experience taking tablets (of any size or shape), making it a novel experience for them. In total, 84% (84/100) of participants had never attempted to swallow a tablet before. The incidence of tablet naivety decreased with age, from 100% in G1 to 68% in G3 participants.

Children under the age of 1 year were required to attempt a single sample only, whereas those aged over 1 year were offered two MTs samples. The overall refusal rate was low, with one refusal recorded for the first sample and eleven for the second. A slightly higher number of refusals was observed for the “low volume” samples compared to the “high volume” samples; however, this difference may reflect the randomisation process rather than a true preference.

The children showed a slight preference for the higher volume of soft food, although both volumes evaluated were considered acceptable. However, due to the pragmatic sample size, the study was not statistically powered to draw definitive conclusions regarding soft food volume preferences.

Yoghurt was the most frequently chosen soft food, with only fifteen children opting to take the MTs mixed in apple sauce. More children mentioned the white coated ‘sprinkles’ were visually prominent in apple sauce than in yoghurt, even after mixing. This observation suggests that the visual appearance of the medicine-food mixture may influence MT acceptability, with less visible formulations (e.g., in yoghurt) potentially being preferred by children.

No adverse events such as vomiting or choking were reported during the administration of the MT sample(s). Although four episodes of coughing were observed by the researcher (yielding swallowability score of 4) these incidents were not clinically significant and were considered unrelated to the MT administration.

Overall swallowability (and thus acceptability) was notably influenced by the frequency of chewing, which was observed in 47% (92/197) of administrations across all age groups. Participants were not instructed to avoid chewing the MT samples unless they specifically asked for guidance beforehand. Chewing is an anticipated behaviour, particularly among children under 5 years of age (Klingmann et al., 2018; Münch et al., 2023). Future studies should advise children who are old enough to understand instructions to avoid chewing the sample.

As part of the participant questionnaire, children aged 4 years and older were asked “If this were a real medicine, would you be willing to take it every day?”. The majority (77%) indicated they would be willing to take the MT formulation daily if regular medication were needed, in the future. However, this proportion decreased slightly to 68% after the second sample administration.

In general, participant-reported outcomes using the hedonic scales were more positive than the CEP scores derived by the researcher. Data from older children, as reported in the questionnaire, were significantly more favourable than the CEP scores. This discrepancy may be attributed to children’s desire to please the parent or researcher during the study visit, leading to a mismatch between the two measures.

Only one parent requested the addition of extra soft food to their 1-year-old child’s first sample, specifically asking for 7.5 mL of yoghurt to be added to the low-volume sample. For children under 1 year, parents selected 15 mL (n = 2) and 20 mL (n = 1) as the ‘age-appropriate’ amount of soft food to be added to their child’s single MT sample, which corresponded to the “high-volume” amounts given to children under 2 years and under 4 years, respectively. This indicates that parents may prefer to add more soft food than the amount initially recommended based on the child’s age. Caution should be exercised when administering medicines with larger volumes of soft food as the entire dose needs to be consumed within an appropriate time frame to achieve the intended clinical effect.

The study successfully enrolled 100 participants across three age groups. Variation in group sizes reflected the voluntary and opportunistic recruitment approach outlined in the study design. This uneven distribution may limit the robustness of comparisons between age groups and should be considered when interpreting the findings. Notably, the group with the largest number of participants was the older group. Video review demonstrated that, although children in this group were cooperative and completed the sampling as instructed, some thought the number of MTs was excessive and expressed their dissatisfaction to the researcher.

Study limitations include assessment of acceptability in a hospital setting on a single occasion, which may not fully reflect the experience of everyday use. The effects of familiarisation or training are also unclear. Future research conducted in home environments over a longer period could provide more realistic insights, for example, regarding the child’s typical behaviour when taking a medicine and their comfort level. Additionally, this study used placebo MTs. The inclusion of an active drug in future MT formulations could alter the taste and, as a result, impact acceptability outcomes (Ranmal et al., 2025).

Although the EMA issued a Letter of Support (European Medicines Agency, 2023) for the original CEP tool (Wargenau et al., 2022), a formal Qualification Opinion has not been granted, indicating that further development and validation are required before regulatory endorsement can be achieved. The tool integrates two key aspects of acceptability - swallowability and palatability - into a single composite measure but does not account for all factors that may influence acceptability in children, such as product appearance (e.g., colour), ease, duration, and frequency of administration, or overall compliance with treatment (European Medicines Agency, 2023).

Palatability assessment from video recordings is inherently subjective, and some variability between reviewers is to be expected. This was evident in the initial inter-rater agreement on palatability assessments, which was 53% (105/200), indicating substantial variability despite prior training. The CEP tool (Wargenau et al., 2022) is designed to accommodate such variability while providing a structured and standardised measure of overall acceptability. To our knowledge, this is the first study to use the CEP tool to evaluate the administration of multiple placebo MTs delivered across several spoonfuls, resulting in prolonged administration times. Longer and more variable administration - particularly among younger children - may have contributed to inter-rater differences by providing more opportunities to observe facial expressions, rather than indicating methodological inconsistency. Administering multiple MTs mixed with soft food is likely to require significantly more time than administering a single dose unit, which may further affect palatability outcomes by allowing observation of a wider range of reactions. Additionally, the limited experience of the two independent reviewers in evaluating facial responses likely contributed to inconsistencies in interpretation during video review. While the CEP tool permits some level of disagreement, extreme disagreement (defined as a ‘contradictory’ outcome) was not allowed in our study. Such disagreement occurred in 23 cases (12%), all of which were reviewed and adjudicated by the Chief Investigator (LB), whose experience in paediatric clinical research and prior use of the CEP tool for video-based assessments ensured consistent scoring. This represents an additional step implemented by our research team compared with the original CEP tool methodology, which allows major disagreements to be included in the palatability results. To maintain consistency, a general overall impression of palatability was applied on a case-by-case basis. To improve consistency and inter-rater reliability in future studies, greater standardisation could be implemented, including detailed scoring guidance, calibration exercises, and structured consensus discussions. Such steps may be particularly important when evaluating formulations with prolonged administration periods or complex dosing schedules.

While the CEP tool has primarily been applied in studies involving single tablets or small numbers of MTs, the present study assessed a broader dosing range (50–135 MTs per sample) reflecting the anticipated dose of the proposed API. By simulating real-world use scenarios, the study provides a more robust and clinically meaningful assessment of acceptability in children. A recent study in 2024 by Münch et al. (2024) utilised a newly adapted version of the original 2022 CEP tool (Wargenau et al., 2022) to evaluate the acceptability of placebo MTs in comparison with three other dosage forms (syrup, oblong tablet, and round tablet) in a cohort of 320 children stratified into 3 age-groups (1-<6 months, 6–12 years, 12–18 years). The number of MTs administered varied with children receiving 3, 11, or 70 MTs depending on their developmental stage. Notably, MTs demonstrated the highest acceptability in the younger age groups when compared to syrup, as assessed by the new CEP tool. In contrast, adolescents showed a preference for oblong tablets. These findings, together with the results of the CAMEO study, strengthen the growing evidence that MTs are a well-accepted SODF for paediatric use, particularly for younger children (Klingmann, 2017).

Texture is an important driver of food acceptance in young children (Werthmann et al., 2015; Chow et al., 2024). In this study, placebo MTs introduced small particulates into soft foods (apple sauce or yoghurt). By varying the soft food volume while keeping the number of MTs constant, the study assessed whether perceived “lumpiness” affected acceptability. Children were generally able to consume the MTs regardless of food volume, suggesting that sprinkle-sized particulates were well tolerated. Future studies should examine a broader range of textures and particulate sizes to further understand their impact on paediatric acceptability.

Our findings may help guide healthcare providers and parents on administering MTs with soft foods, including practical advice on acceptable soft food volumes. Future work could also focus on developing and evaluating educational resources, such as written instructions or videos, to support safe and effective use of MTs in paediatric populations.

Future research should address the current study’s limitations through stratified recruitment, objective palatability assessments, and potential refinements to the CEP tool. Incorporating longitudinal, home-based evaluations would provide valuable real-world insights and generate patient-centred evidence to inform paediatric formulation development and provide practical guidance for families administering MTs minitablets to young children. Further validation of the CEP tool across a broader range of oral formulations, paediatric age groups - including children receiving long-term medication - and real-world administration settings is warranted to confirm its robustness and generalisability. In parallel, the development of a comprehensive user guide with exemplar scenarios, alongside exploration of digital enhancements such as automated facial expression analysis software, could strengthen reliability and facilitate wider adoption. Importantly, future iterations of acceptability assessment tools should continue to be co-developed with children and young people to ensure their relevance, usability, and child-centred design (Reidemeister et al., 2023).

## Data Availability

The original contributions presented in the study are included in the article/Supplementary Material, further inquiries can be directed to the corresponding author.

## Abbreviations

AH, Alder Hey Children’s NHS Foundation Trust; API, Active pharmaceutical ingredient; CAMEO, Children’s Acceptability of Minitablets mixEd with food; CEP, Composite endpoint; CI, Chief investigator; CRF, Clinical research facility; %EA, Percentage exact agreement; EMA, European Medicines Agency; FDA, U.S. Food and Drug Administration; G1, Age group 1; G2, Age group 2; G3, Age group 3; MT, Minitablet; NHS, National Health Service; NIHR, National Institute for Health and Care Research; PMRU, Paediatric Medicines Research Unit; R1, Researcher 1; R2, Researcher 2; S1, Sample 1 (1^st^ sample given); S2, Sample 2 (2^nd^ sample given); SmPC, Summary of Product Characteristics; SODF, Solid oral dosage form; YPAG, Young Persons’ Advisory Group.
