# Supplementary material for: Acceptability of minitablets in soft food. A randomised cross-over study in children
Source: Front Pharmacol. 2026 Jan 5;16:1702183. doi: 10.3389/fphar.2025.1702183 (PMC12812957; doi:10.3389/fphar.2025.1702183)
Supplement: Supplementary file 1 [file Supplementaryfile1.docx]

Supplementary Material

**Composite endpoint tool for acceptability assessment used in the CAMEO study**

**Table S1 Scoring criteria for swallowability**

| **Score** | **Observation** | **Interpretation** |
| --- | --- | --- |
| 1 | Completely swallowed | Full sample consumed without chewing & no residual minitablets found in the mouth. |
| 2 | Partially swallowed | Sample chewed and/or there are residuals of the minitablets found in the mouth (at least 80% must have been consumed). |
| 3 | Spat out | Sample fully or partially spat out. |
| 4 | Swallowed the wrong way | Cough may have been caused. |
| 5 | Refused to take | Less than 80% consumed.  Immediate refusal made or part of the minitablet sample attempted but then refused (study activity stopped). |

Table modified from Wargenau et al. (2022)

**Table S2 Palatability scoring criteria based on video documentation per rater**

| **Score** | **Assessment** | **Interpretation** |
| --- | --- | --- |
| 1 | Pleasant | **Positive hedonic pattern:**  Tongue protrusion, smack of mouth and lips, finger sucking, corner of the mouth elevation |
| 2 | Neutral | Neutral mouth and body movements, and face expression |
| 3 | Unpleasant | **Negative aversive pattern:**  Gape, nose wrinkle, eye squinch, frown, grimace,  head shake, arm frail |

Table reproduced from Wargenau et al. (2022)

**Table S3 Combined rater palatability assessment**

| **Scoring of Rater 1** | **Scoring of Rater 2** | | |
| --- | --- | --- | --- |
|  | Pleasant | Neutral | Unpleasant |
| Pleasant | Pleasant | Pleasant | Contradictory* |
| Neutral | Pleasant | Neutral | Unpleasant |
| Unpleasant | Contradictory* | Unpleasant | Unpleasant |

*Contradictory result = extreme disagreement
Table modified from Wargenau et al. (2022)

**Table S4 Assessment of acceptability as composite endpoint**

| **Palatability (combined rater)** | **Swallowability Score** | | |
| --- | --- | --- | --- |
|  | 1 | 2 | ≥3 |
| Pleasant | High | Good | No |
| Neutral | Good | Low | No |
| Unpleasant | Low | No | No |
| Contradictory | Good | Low | No |

Table reproduced from Wargenau et al. (2022)
