# Supplementary material for: Acceptability of minitablets in soft food. A randomised cross-over study in children
Source: Front Pharmacol. 2026 Jan 5;16:1702183. doi: 10.3389/fphar.2025.1702183 (PMC12812957; doi:10.3389/fphar.2025.1702183)
Supplement: Supplementary file 2 [file Supplementaryfile2.docx]

Supplementary Material

**Figure S5: Number of participants by age (years), stratified by gender**
